# Perception of Air Pollution in the Jinchuan Mining Area, China: A Structural Equation Modeling Approach

**DOI:** 10.3390/ijerph13070735

**Published:** 2016-07-21

**Authors:** Zhengtao Li, Henk Folmer, Jianhong Xue

**Affiliations:** 1 Department of Industry and Economics, School of Economics and Finance, Xi’an Jiaotong University, No. 74, the West Yanta Road, Xi’an 710061, China; 2 Department of Economic Geography, Faculty of Spatial Sciences, University of Groningen, Landleven1, Groningen 9747 AD, The Netherlands; h.folmer@rug.nl; 3 Department of Agricultural Economics, College of Economics and Management, Northwest A & F University, 3 Taicheng Road, Yangling 712100, China; xuej@nwsuaf.edu.cn

**Keywords:** perception of air pollution, environmental knowledge, structural equation model (SEM), latent variable, China

## Abstract

Studies on the perception of air pollution in China are very limited. The aim of this paper is to help to fill this gap by analyzing a cross-sectional dataset of 759 residents of the Jinchuan mining area, Gansu Province, China. The estimations suggest that perception of air pollution is two-dimensional. The first dimension is the perceived intensity of air pollution and the second is the perceived hazardousness of the pollutants. Both dimensions are influenced by environmental knowledge. Perceived intensity is furthermore influenced by socio-economic status and proximity to the pollution source; perceived hazardousness is influenced by socio-economic status, family health experience, family size and proximity to the pollution source. There are no reverse effects from perception on environmental knowledge. The main conclusion is that virtually all Jinchuan residents perceive high intensity and hazardousness of air pollution despite the fact that public information on air pollution and its health impacts is classified to a great extent. It is suggested that, to assist the residents to take appropriate preventive action, the local government should develop counseling and educational campaigns and institutionalize disclosure of air quality conditions. These programs should pay special attention to young residents who have limited knowledge of air pollution in the Jinchuan mining area.

## 1. Introduction

Many studies have shown that air pollution is a major public health concern for a large part of the world’s population (see inter alia [1,2,3]). The adverse health effects of exposure to, especially, particulate matter (PM) and sulfur dioxide (SO_2_), range from respiratory illnesses [4,5] and cardiovascular illnesses [6,7], to lung cancer [8,9] and premature death [10].

The situation in China is worse than in many other parts of the world. Economic development has greatly increased energy consumption, mining, and construction in recent decades [11,12] leading to serious air pollution in many urban, industrial and mining areas. The main air pollutants include nitrogen dioxide (NO_2_), ozone (O_3_), sulfur dioxide (SO_2_) and particulate matter (PM) [13,14].

Jinchuan, which is located in the western province of Gansu, is one of the ten cities with the most serious air pollution in China [15]. It has the largest nickel resource in the country and mining and smelting industries dominate its economy and make a substantial contribution to its development. However, the two industries also create serious environmental problems, including industrial solid waste and soil, water and air pollution. The main air pollutants include suspended particles, sulfur dioxide, chlorine gas and carbon dioxide [15,16,17]. An important question is if, and to what extent, the residents of Jinchuan have adopted their behavior to mitigate the health impacts caused by air pollution (averting behavior) in their home region.

Most studies on averting behavior related to (air) pollution link averting behavior to objective measures of emissions. However, such an approach ignores the fact that averting behavior presupposes contextual knowledge of the pollution in question and related hazards [18,19,20]. These authors [18,19,20] argued that not only factual information about pollution is applied in decision making related to averting behavior but also environmental knowledge and perception of the situation. Another important reason to consider perception rather than relying on factual information is that laboratory experiments have frequently indicated that individuals tend to under-estimate high risk events and over-estimate small risk events [21,22].

Analyses of the impacts of perception on behavior usually do not distinguish between perception of the *occurrence of pollution* and perception of its *health risks* (which is subjective probability based on an individual’s personal judgment and is different from objective probability which is calculated from observed frequencies [23]). The two kinds of perception are usually taken together and the combination is analyzed. However, *perception of the probability of suffering from an illness as a result of pollution* is different from *the perception of pollution per se.* There is not an automatic link between perception of the probability of occurrence of pollution and perception of the likelihood to incur an illness [24,25]. In particular, the former is a prerequisite for the latter, which in its turn affects averting behavior aimed at reducing the health risk. Ignoring the difference may lead to loss of information and to biased estimation results. Specifically, an insignificant impact of risk perception on averting behavior could actually be a consequence of a lack of perception of pollution per se [18,26]. The distinction between both types of perception is also relevant for educational campaigns aimed at stimulating averting behavior [26]. Meaningful campaigns should target the right kind of perception. 

The distinction between perception of pollution as such and perception of the likelihood to incur an illness is especially relevant in the case of a country, such as China, where information about pollution is often not disclosed or not freely available [27]. Chinese citizens who seek information about pollution are often considered “troublemakers” by the Government and corporate officials [28] and only inessential information tends to be disclosed to the public to comply with regulations [29]. Furthermore, the disclosed information may not be entirely accurate in order to mask or falsify actual environmental performance [30]. The above conditions definitely apply to the city of Jinchuan, which is located in a remote area and critically depends on its mining and smelting industries. 

Perception of environmental hazards including perceived health risks has become a multi-disciplinary field of research including psychology, economics, geography, health sciences, anthropology and sociology. Studies on the perception of a wide variety of environmental hazards have been undertaken including forest fire [31], drinking water [32], pesticides [33,34], earthquakes [35,36] and nuclear power [37]. Bickerstaff and Walker [1] and Howel et al. [38] focused on the perception of the occurrence of air pollution and [39,40,41] on the health risk of air pollution. Richardson et al. [42] analyzed the economic health costs of exposure to wildfire smoke and [25] analyzed the interrelations between odorous air pollution levels, perceived pollution, health risk perception, annoyance and health symptoms. 

The main objective of this paper is to analyze the determinants of the perception of the intensity and hazardousness of air pollution in the Jinchuan mining area, Gansu Province, China, based on a cross-sectional sample of 759 respondents. It makes the following contributions to the literature: First, a better understanding of the perception of health risks in the Jinchuan mining area which is China’s “nickel capital” [15]. Second, it empirically identifies the dimensions of perception of pollution in the study area. Third, it contributes to a better understanding of the effect of people’s environmental knowledge on their perception of (air) pollution and vice versa. Fourth, it estimates the effects of socio-demographic characteristics on environmental knowledge and perception of air pollution. Finally, it is intended to stimulate of national and local governments to develop counseling and educational campaigns in the short run and air pollution abatement and management programs in the long run. 

This paper is organized as follows. Section 2 presents the conceptual model. It defines the endogenous variables perception of air pollution and environmental knowledge, specifies the interdependencies among the endogenous variables mutually as well as the impacts of the exogenous variables on the endogenous. Section 3 describes the survey and sampling method and the econometric methodology (SEM). Section 4 presents the empirical results. Section 5 discusses the study’s findings and Section 6 presents the conclusions. 

## 2. Conceptual Model

In this section, the conceptual model (in the literature also denoted as the theoretical model) is presented. It consists of the theoretical definitions of the variables (nomenclature), the rationale for their inclusion in the model, the relationships among the endogenous variables, and the impacts of the exogenous variables on the endogenous variables. The conceptual model thus forms the hypotheses, which are tested in Section 4 (Empirical Results). It is based on a literature review, on consultation with experts on environmental problems—notably air pollution and protection against it in Jinchuan- and on intuition. (See inter alia [43] for details about the conceptual model as a set of hypotheses and its role in empirical analysis.).

Before going into detail, we note that since perception of air pollution is an aspect of the perception of its health risks, the literature review relates to both types of perception, especially because the literature on perception of (air) pollution per se is small.

**Figure 1:**
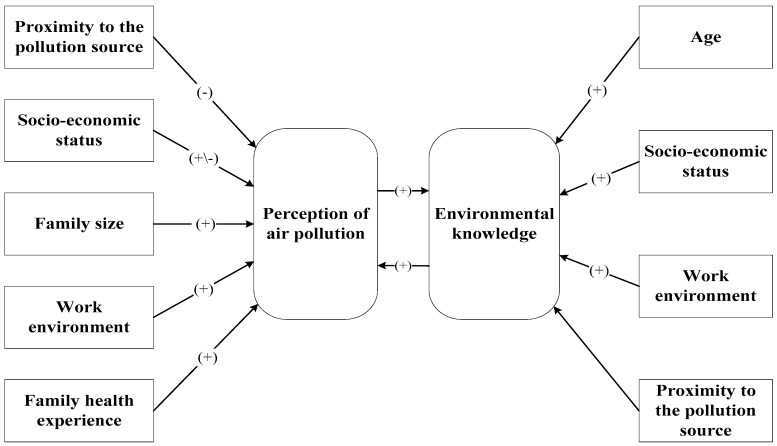
The Perception of air pollution-Environmental knowledge (PAP-EK) model. **Note:** Within the brackets are the expected signs.

The Perception of air pollution-Environmental knowledge (PAP-EK) conceptual model is summarized in Figure 1. It contains two endogenous variables, **Perception of air pollution (PAP)** and **Environmental knowledge (EK)**, and the exogenous variables **Age**, **Proximity to the pollution source**, **Work environment**, **Socio-economic status** and **Family health experience**. In Figure 1, an arrow denotes the direction of a hypothesized relationship (impact) between an exogenous and an endogenous variable or between the two endogenous variables. The endogenous variables **PAP** and **EK** and their relationships are discussed first. Next, the effects of the exogenous variables, i.e., **Socio-economic status**, **Age**, **Family size**, **Family health experience**, **Proximity to the pollution source** and **Work environment** on **PAP** and **EK**, are discussed.

### 2.1. Endogenous Variables

**Figure 2:**
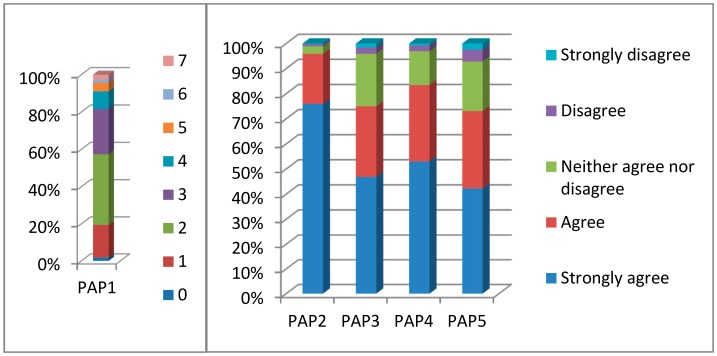
Frequency distribution of the indicators of the **Perception of air pollution**. **Notes**: **PAP1**: How many days a week did you perceive the air in Jinchuan city to be heavily polluted last year? **PAP2:** Jinchuan’s air pollution increases the possibility of suffering from respiratory illnesses. **PAP3:** Jinchuan’s air pollution increases the possibility of suffering from cardiovascular illnesses. **PAP4:** Jinchuan’s air pollution increases the possibility of suffering from lung cancer. **PAP5:** Jinchuan’s air pollution increases the possibility of suffering from death. Source: Author’s survey

**Perception of air pollution (PAP)*.*** The main dependent variable of the PAP-EK model is **PAP**. Sjöberg et al. [44] defined **PAP** as the subjective assessment of its intensity and the concern with the consequences of exposure to it. Two dimensions are contained in this definition: (i) perception of intensity of air pollution; and (ii) perception of hazardousness of the pollutants. The first dimension is measured by the question: “What is your perception of the average number of days per week that Jinchuan’s air was heavily polluted during the past year?” (**PAP1**) The second dimension was measured by presenting four major types of health problems related to air pollution to the respondents (**PAP2–5**). They were asked to indicate to what extent they perceived Jinchuan’s air pollution as being responsible for inducing health problems. The four major health problems were respiratory diseases, cardiovascular diseases, lung cancer and death (see Figure 2 for details). 

The observed variables are taken as indicators of a latent variable, **PAP**. Latent variables relate to phenomena that are supposed to exist but cannot be directly observed. The reason is that they are theoretical constructs that do not correspond to anything that can be measured, or that observations of the phenomena concerned are measured with error. Examples of latent variables are welfare, happiness and intelligence. A latent variable is given empirical meaning by way of correspondence statements that link a latent variable to its indicators, which are observed variables. Observed variables such as age, income and education, possess direct empirical meaning. For instance, the latent variable intelligence is given empirical meaning by means of intelligence tests. For further details on latent variables, see amongst others [45,46,47,48]. (In this paper, there are three latent variables: **PAP, EK** and **Socio-economic status**). As a first step, we assume that all five observed variables are indicators of a single latent variable, **PAP**. 

**Figure 3:**
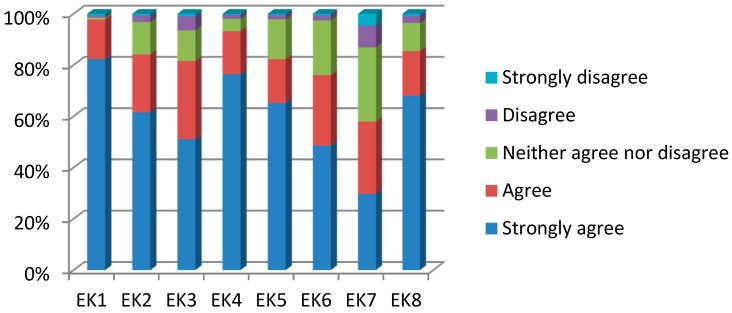
Frequency distribution of the indicators of **Environmental knowledge**. **Notes**: **EK1:** Jinchuan suffers from air pollution. **EK2**: Jinchuan suffers from industrial solid waste. **EK3**: Jinchuan suffers from water pollution. **EK4**: Environmental issues in Jinchuan are mainly caused by local industrial activities. **EK5**: Sulfur dioxide is one of the main air pollutants of Jinchuan. **EK6**: Suspended particles is one of the main air pollutants of Jinchuan. **EK7**: Carbon dioxide is one of the main air pollutants of Jinchuan. **EK8**: Chlorine gas is one of the main air pollutants of Jinchuan. Source: Author’s survey

**Environmental knowledge (EK).** Berkes et al. [49] defined **EK** as one’s body of knowledge of the interdependency between human society and its natural environment. Based on the air quality conditions in Jinchuan, the respondents’ knowledge of air pollution was measured by means of eight items. The first four are related to general environmental issues and their causes, and the remainder are related to the main air pollutants in the area (see Figure 3). Note that the notion of environmental knowledge is related to, though different from, notions such as environmental awareness and beliefs. De Lavega [50] pointed out that environmental awareness is the concern and sensitivity towards the environment and its problems. Madsen [51] showed that awareness is a driving force of knowledge accumulation. The difference between knowledge and belief depends on the degree of uncertainty. When uncertainty diminishes, belief gradually turns into knowledge [52,53].

**PAP** and **EK** are assumed to interact. First, as explained by [54]. **EK** is an important determinant of **PAP**. A positive impact is expected: better knowledge improves perception. Evidence for this hypothesis can be derived from [32] who found that better knowledge of a pesticide increased the perception of the risk following exposure to it. Similar results were found by [55,56]. Secondly, **PAP** is assumed to stimulate **EK.** A positive effect is expected: the more pollution one perceives, the more knowledge of it will be collected [57,58].

Note that the interaction between **PAP** and **EK** may be subject to self-confirmation bias, i.e., the tendency that people look for evidence that confirms their beliefs and overlook the evidence that goes against them [59]. For instance, [60] conducted research on tuberculosis in the UK and found that perception of tuberculosis induced people to look more for information that confirmed their beliefs than for information that denied them. If there is self-confirmation bias, people who perceive air pollution will: (i) expand their environmental knowledge; and (ii) select environmental knowledge that confirms their beliefs and overlook evidence against them. An insignificant impact of **PAP** on **EK** knowledge is evidence against self-confirmation bias. The issue is discussed further in Section 4.2.

### 2.2. Exogenous Variables

**Age (AGE)*.* Age** is assumed to have a positive impact on **EK**. The rationale is that older people have lived longer in Jinchuan than younger people and thus have more experience with, and better knowledge of, Jinchuan’s mining and smelting industries. Support for the hypothesis can be derived from [61,62]. An indirect impact of **Age** on **PAP**—via **EK**—is hypothesized.

**Proximity to the pollution source (PPS). PPS** is assumed to have a direct impact on **EK**. The number of studies on the relationship between **PPS** (smelting plants) and **EK** is very limited. An exception is [63], who studied noise pollution in Zahedan, Iran. The author did not find evidence of differences in peoples’ knowledge based on distance from the pollution source. Given the weak evidence from the literature, an expected sign is not specified.

A negative impact of **PPS** on **PAP** is postulated because respondents who live further away from the smelting plants are less exposed than those who live nearby. Evidence can be derived from [1,38]. Three categories are distinguished:

(1) SAP (Nearby smelting plants, serious air pollution);
(2) MAP (medium air pollution); and
(3) LAP (far away from pollution source, light air pollution).

**Work environment (WE)*.*** A positive impact of **WE** on **EK** is assumed. Since it is the source of Jinchuan’s environmental issues, people working in the Jinchuan Mining Company (JMC) are expected to have better knowledge of Jinchuan’s environmental issues than non-JMC individuals. In particular, JMC employees, especially miners and smelter workers, are expected to know more about the input and output of the smelting process than non-JMC individuals. Evidence for the hypothesis can be drawn from [32,64]. 

**Work environment** is also hypothesized to have an impact on **PAP**. That is, the JMC employees, especially the ones who work in the mine or smelter, are expected to have higher perception levels than non-JMC individuals. The rationale is that their work places are the most seriously polluted. Support for the hypothesis can be derived from [18,65]. Three **WE** classes are distinguished:

(1) MS (miners and smelter workers of JMC);
(2) NMS (people who are JMC employees, but not miners or smelter workers); and
(3) NMC (non-JMC individuals) which is the base case.

**Socio-economic status (SES)*.* SES** is commonly conceptualized as an individual’s standing in society [66]. It is measured here by means of the observed variables **Educational** **Attainment** (**EDU**) and **Household Net Income (IN)**. **SES** is assumed to positively impact **EK**. This hypothesis is supported by [67,68,69]. A direct impact of **SES** on the **PAP** is assumed. 

The literature is ambiguous about the signs of the impact. A positive relationship was found by [70,71,72]. On the other hand, [73] analyzed data from a national survey in Canada and found that **SES** was negatively correlated with perception of health risk. Because of the opposing views, no expected sign is a priori assigned. **EDU** is measured as the highest degree obtained and **IN** as after tax household income (see Table 1 for details).

**Family size (FS)*.*** A positive impact of **FS** on **PAP is** assumed, because in larger families more people are exposed than in smaller families. This assumption is supported by [72,74]. No direct impact of **FS** on **EK** is assumed. **FS** is defined as the number of family members who live in the same house. 

**Family health experience (FHE)*.* FHE** is assumed to have a positive impact on **PAP**. This assumption is supported by [38,75]. An indirect impact of **FHE** on **EK**—via **PAP**—is hypothesized. **FHE** is measured by means of a dichotomous variable which takes the value 1 if the respondent, or one or more of their family members, has been hospitalized for cardiovascular or respiratory disease. 

## 3. Methods

### 3.1. Sampling and Data Collection

A survey was conducted in July and August 2012 in the Jinchuan mining area. In order to capture air pollution characteristics from the Jinchuan mining area, stratified sampling (see [76] for details) was applied based on the degree of pollution (severely, moderately and lightly polluted areas) which corresponds to the distance to the pollution source (smelting plants). Within each stratum, households were randomly sampled proportional to the stratum size relative to the population size. Per hundred households, one to two households were selected. Interviewees, who were permanent Jinchuan residents with Jinchuan “Hukou”, were family heads, usually the husbands. (A Hukou is a record in China’s household registration system. It identifies a person as a resident of a village, town or city and includes information such as name, parents, spouse, and date of birth. A Hukou serves as a domestic passport and regulates population distribution, rural-to-urban migration and eligibility to public services.) The minimum age for respondent in the survey was twenty-one years; no upper age limit was imposed. The sample consisted of 800 family heads.

The questionnaire was administered through face-to-face interviews. Interviewers were students of Gansu Non-ferrous Metallurgy College in Jinchuan, who were familiar with environmental issues in Jinchuan and had mastered the local language. To reduce interviewer variability impact on information gathering, all interviewers underwent a training program to ensure that they had the skills and knowledge to correctly carry out interviews. The questionnaire contained questions about a respondent’s environmental knowledge, perception of air pollution and socio-demographic characteristics. A pilot survey was conducted to test each question and to revise it, if necessary. Each interview took approximately an hour.

### 3.2. Structural Equation Modeling (SEM)

The conceptual PAP-EK model is estimated as a structural equation model with latent variables (SEM) for the following reasons. First, the conceptual model contains two interacting endogenous variables (see Figure 1 and Equation (4) below). In particular, Figure 1 hypothesizes interaction between **EK** and **PAP**. To model interacting endogenous variables, simultaneous-equations models are required (see inter alia [77,78]). Secondly, the model contains both latent and observed variables. Both types of variables can be simultaneously handled by SEM because it is made up of: (i) two measurement models that represent the correspondence statement between the latent variables and their indicators; and (ii) a structural model that specifies the relationships between the latent variables (some or all of which may be identical to their observed indicators (see [77] and the references therein). (It is possible to combine the two measurement models, see [78]. Furthermore, means and intercepts can be included in the system. However, they are not included in this paper because all variables are standardized.)

Equation (1) is the structural model:

(1) η=Βη+Γξ+ζ

where η and ξ are the (m × 1) and (n × 1) vectors of latent endogenous and exogenous variables, respectively. The (m × m) matrix Β contains the structural relationships among the latent endogenous variables. The impacts of the exogenous latent variables on the endogenous latent variables are given by Γ**,** which is an (m × n) matrix. ζ is a random (m × 1) vector of errors with covariance matrix Ψ(m×m). The covariance matrix of ξ is Φ(*n* ×n).

Equations (2) and (3) are the measurement models that present the relationships between the latent variables and their indicators (correspondence statements):

(2) $$y=\Lambda_{y}\eta +\varepsilon$$

(3) $$x=\Lambda_{x}\xi +\delta$$

where **y** and **x** are the (p × 1) and (q × 1) vectors of observed endogenous and exogenous variables, respectively. The (p × m) and (q × n) matrices $\Lambda_{y}$ and $\Lambda_{x}$ specify the relationships (loadings) between **y** and **x** and their corresponding latent variables ***η*** and **ξ**, respectively. ε and δ are the measurement errors with covariance matrices $\Theta_{\varepsilon }\left(p\times p\right)\text{ and }\Theta_{\delta }\left(q\times q\right)$, respectively. (Note that a directly observed variable can be straightforwardly handled in the SEM framework by defining it identical to its corresponding latent variable and specifying an identity relationship in the measurement model.)

Estimation can be done by means of the software packages Lisrel 8, OpenMx (in R), AMOS and Mplus. Lisrel 8, applied in this paper, is probably best known (for details, see [77]). Lisrel 8 also provides information on identification and various test statistics including overall goodness of fit statistics, z-statistics for each coefficient and $R^{2}s$ for the measurement model equations and the structural equations. Moreover, it provides modification indices that can be used to improve the model fit by freeing incorrectly constrained or fixed parameters.

## 4. Empirical Results

### 4.1. Descriptive Statistics

The number of dropout as a result of incomplete questionnaires was 41 (5.12 percent) which gives a dataset of 759 observations. There was no evidence of non-random drop out. 

Descriptive statistics are presented in Table 1, and Figure 2 and Figure 3. The distribution of the socio-economic characteristics in Table 1 is in line with the population distribution of Jinchuan [79]. As for intensity of air pollution (**PAP1**), Figure 2 shows that the majority (62.1%) of the respondents perceived medium pollution (2 or 3 days a week), 18.3% heavy pollution (4 or more days a week), and the rest (19.6%) light pollution (0 or 1 days a week). The other indicators in Figure 2 measure perception of hazardousness as potential causes of health problems, specifically cardiovascular illnesses, respiratory illnesses and death (**PAP2–PAP5**). A five-point scale was used with 1 indicating weak perception and 5 indicating strong perception. Figure 2 shows that the vast majority (over 70%) of the respondents perceived the pollution to be highly or moderately hazardous.

Figure 3 shows how knowledgeable the respondents are about Jinchuan’s environment. For each statement a five-point scale ranging from 1 (strongly disagree) to 5 (strongly agree) was used. The first three statements (**EK1**–**EK3**) measure knowledge of general environmental issues in Jinchuan. As can be seen from Figure 3, the vast majority of the respondents strongly agree or agree that air pollution, industrial solid waste, and water pollution are serious environmental issues. With regard to the causes (measured by **EK4**), almost all of the respondents (93.2%) acknowledge that local industries are the main source of Jinchuan’s environmental problems. The last four statements (**EK5**–**EK8**) test the respondents’ knowledge of the main air pollutants. The results show that chlorine gas is acknowledged as the main pollutant (85.5%), followed by sulfur dioxide (82.3%), suspended particles (76%) and carbon dioxide (57.9%).

### 4.2. The Estimated SEM

The hypothesized relationships in the Conceptual Model, summarized in Figure 1, are presented in Equation (4), the structural component of the SEM. It reads:


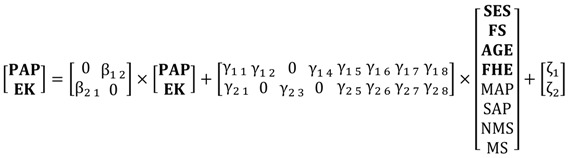
(4)

The measurement models, which are not given in Section 2, are presented in Equations (5) and (6):

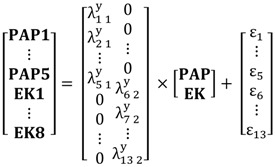
(5)

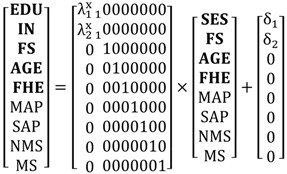
(6)

Note: To make the SEM identified and the parameters interpretable, the variances of the latent variables are fixed at 1; see [77] for details.

The dataset contains several ordinal and dichotomous variables, in particular, the indicators of **PAP** and **EK**. Moreover, the distributions of these indicators are highly skewed and non-normal (see Figure 2 and Figure 3). For these reasons, the PAP-EK model was estimated by WLS based on the matrix of polychoric correlations [77].

**Figure 4:**
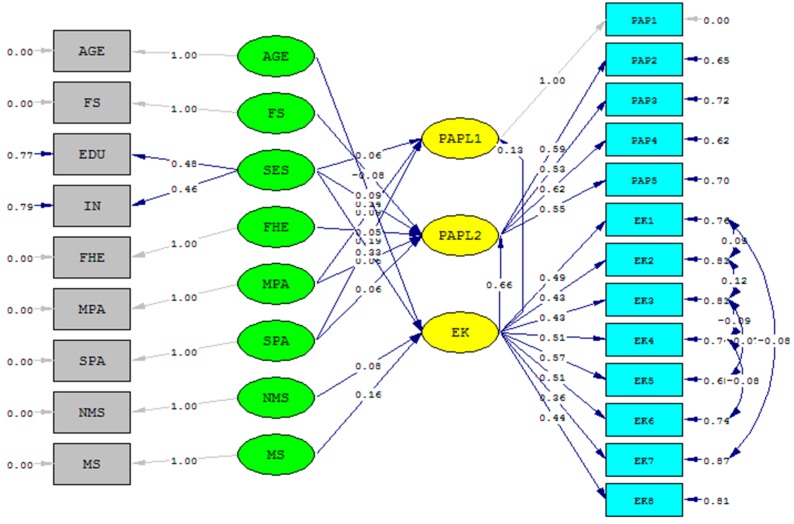
Path diagram of the Final model.

First, the complete conceptual model, as specified in Equations (4)–(6) (denoted Initial model below) was estimated. The Initial model contained several insignificant variables. They were eliminated step-by-step where, at each step, the variable with the highest *p*-value was deleted. A path diagram of the Final model is presented in Figure 4.

Comparison of the overall goodness of fit statistics between the Initial and the Final model indicates whether the insignificant variables were correctly deleted. The overall goodness of fit statistics, presented in Table 2, are the Goodness-of-Fit Index(GFI), the χ^2^/DF (DF denoting degrees of freedom), the Comparative Fit Index (CFI), the Incremental Fit Index (IFI), the Adjusted Goodness-of-Fit Index (AGFI) and the Root Mean Square Error of Approximation (RMSEA) (see [77,80] for details). The almost negligible differences between the goodness of fit statistics of the Initial and Final model support the deletion of the insignificant variables. Particularly, GFI and AGFI are equal while the χ^2^/DF, the RMSEA, IFI and CFI slightly improved.

Before turning to the estimated models, it should be noted that to facilitate comparison of coefficients (i.e., effects), standardized coefficients (beta coefficient) were estimated. A standardized coefficient represents the standard deviation change in the dependent variable for a one standard deviation change in the corresponding explanatory variable. Below the estimated measurement models are first discussed and then the structural model. 

The measurement models are shown in Table 3. It contains the standardized coefficients (loadings), standard errors and reliabilities ($R^{2}s$), i.e., the proportion of variation of an indicator explained by its corresponding latent variables. 

In the Initial model the indicator **PAP1** has very low reliability (0.03) and its coefficient is substantially smaller than the coefficients of the other indicators (**PAP2**–**PAP5**). This is an indication that **PAP1**–**PAP5** should not be taken as indicators of *one* underlying latent variable, **PAP**, but instead, that there are two latent perception variables: (i) **Perceived intensity of air pollution (PAPL1)**; and (ii) **Perceived hazardousness of air pollution (PAPL2).** They are defined as the subjective assessment of the frequency of occurrence of heavy air pollution and of the hazardousness of the main air pollutants, respectively. To distinguish the latent variables from their indicators, “L” is added to their labels.

**PAPL1** is identical to its sole indicator **PAP1** whereas **PAPL2** is measured by the indicators **PAP2**–**PAP5**. The distinction is in line with [44] who introduced the distinction and with [18] who further developed both dimensions and provided empirical evidence for the distinction. The partitioning of **PAP** into two separate latent variables is supported by the modification index of **PAP1** as a possible indicator of **PAPL2** which is very low (0.39). (The modification indices are available from the first author upon request.) Table 3 shows that **PAP2**–**PAP5** are adequate indicators of the latent variable **PAPL2** with significant loadings and satisfactory reliabilities. The table furthermore shows that the indicators of the latent variables EK and **SES** in the Final model significantly load on their corresponding latent variables and that the reliabilities are satisfactory. 

Table 4 shows the structural model. It presents the standardized coefficients, standard errors and $R^{2}s$. As hypothesized in the conceptual model, **EK** has a positive and significant impact on **PAPL1** (0.13) and **PAPL2** (0.66), respectively. However, the reverse effect (0.22) was not significant in the Initial model and therefore was deleted. A possible explanation for the insignificance is that the suffocating and pungent odor of sulfur dioxide and chlorine gas, which are the main directly observed air pollutants in the area, is sufficient evidence of air pollution. The persistence of the odor renders further knowledge acquisition redundant. Furthermore, the insignificance of both perception variables on **EK** indicates absence of self-confirmation bias, as there is no evidence for the first step specified in Section 2. In the Initial model the distance effect (MAP (−0.02) and SAP (0.05)) is highly insignificant which is in line with [63]. Therefore these variables are not considered in the Final model.

The discussion below is primarily based on the Final model. The exogenous variables of **EK** are discussed first and next those of **PAPL1** and **PAPL2**. The Final model shows that **SES** positively and significantly influences **EK** (0.33), which is in line with [67,68,69]. As in [62], **Age** has a positive and significant impact (0.09) on **EK** indicating that living longer in Jinchuan increases knowledge about its mining and smelting industries and related environmental issues. **WE**, measured by the dummies MS (miners and smelter workers) and NMS (JMC employees, but not miners or smelter workers) with non-JMC individuals as the reference case is also an important determinant of **EK**. The impacts are 0.16 and 0.08, respectively. Apparently, miners and smelter workers of the mining company have better knowledge of Jinchuan’s environmental issues than the non-JMC employees. This result is in line with [32,64]. 

The main results regarding the perception variables are as follows. In addition to its indirect effect via **EK**, **SES** directly and significantly impacts **PAPL2** (0.14). Its direct impact (0.06) on **PAPL1** is also positive, though marginally significant. The difference in impacts on **PAPL2** and **PAPL1** is probably caused by the fact that intensity is more easily observable than hazardousness of pollutants which requires acquisition and processing of information. **FS** has a negative impact (−0.08) on **PAPL2** which is in contrast to the Conceptual model. A possible explanation is that perception of air pollution per se is linked to risk perception. Larger families have larger social networks, which offers opportunities for burden sharing [81,82] via cooperation or the use of shared resources [83,84]. **FHE** has a positive impact (0.05) on **PAPL2**, as hypothesized in the Conceptual model. This result is in line with [38,74]. As expected, and in line with [1,85], living in severely and moderately polluted areas increases both **PAPL1** (MAP (0.09) and SAP (0.19)) and **PAPL2** (MAP (0.06) and SAP (0.06)), although the latter impact is marginally significant. Finally, in the Initial model, the impact of **WE** on **PAP** (NMS (−0.03) and MS (0.06)) was insignificant. This could be because of employment dependency, which tends to mask the negative impacts of variables such as **WE** [73]. Since no significant **WE** effect was discerned in the Initial model, **WE** was not included in the **PAPL1** and **PAPL2** equations in the Final model. 

Table 5 presents the standardized total and indirect effects of all (endogenous and exogenous) variables on all endogenous variables in the Final model. An indirect effect represents the effect of a variable on another variable through intervening endogenous variables [77]. The total effect is the sum of the direct and indirect effects. Table 5 shows that serious air pollution (SAP), **EK**, **SES** and medium air pollution (MAP) are the four most important predictors of **PAPL1** with total effects of 0.19, 0.13, 0.10, and 0.09, respectively. **Age**, mine and smelter workers (MS) and other JMC employees (NMS) also positively influence **PAPL1** with total effects of 0.01, 0.01 and 0.02, respectively. The effect of NMS, however, is not significant.

Table 5 furthermore shows that **EK** has the largest positive total effect (0.66) on **PAPL2**, followed by **SES** with a total effect of 0.35. **Age** and **FHE** also positively and significantly influence **PAPL2** with total effects of 0.06 and 0.05, respectively. **FS** negatively and significantly influences **PAPL2** with a total effect of −0.08. Although they have no direct effects on **PAPL2**, NMS and MS positively influence it through **EK**, with total effects of 0.05 and 0.10, respectively. The effect of NMS, however, is not significant.

**SES** is the most important determinant of **EK** with a total effect of 0.33. NMS and MS positively impact **EK**, but only MS is significant with a total effect of 0.16. Next is **Age** (0.09).

## 5. Discussion 

In this paper, a structural equation model (SEM) was applied to analyze perception of air pollution in the Jinchuan mining area, Gansu Province, China, based on a cross-sectional sample of 759 respondents. To the best of the authors’ knowledge, this is the first paper to address this issue in China.

The literature on air pollution does usually not distinguish between the perception of the occurrence of air pollution and the perception of the probability of suffering from an illness caused by exposure to it (risk perception). However, ignoring the difference may lead to loss of information and biased estimation results. The distinction between both types of perception is also relevant for educational campaigns aimed at stimulating averting behavior. Adequate campaigns should target the right kind of perception. 

It was shown that there are two types of perception: perceived intensity and perceived hazardousness of the pollutants. Both types are influenced by environmental knowledge. However, there are no reverse effects from perception to environmental knowledge. The first type of perception is furthermore influenced by socio-economic status and proximity to the pollution source; the latter by socio-economic status, family health experience, family size and proximity to the pollution source. Environmental knowledge is influenced by socio-economic status, age and work environment. 

The main finding is that virtually all respondents (97.8%) acknowledged that Jinchuan’s air is heavily polluted. In particular, more than 70% perceived high intensity and hazardousness of air pollution despite the fact that public information on air pollution and its health impacts is not freely available. Since environmental knowledge positively impacts on the perception of air pollution, information on air quality should be widely disclosed to enable the residents to take action to reduce exposure and health risks. This applies especially to young people and those with low education who have limited knowledge. Furthermore, even though people may be knowledgeable of air pollution in the region in general, they may be unable to collect information about its day-to-day severity. Therefore, in combination with the weather forecast, information on local air quality conditions could be communicated and risk mitigating actions such as reducing or abandoning outdoor activities could be recommended. The finding that environmental knowledge among older age groups is better than among younger people implies that more attention should be paid to young people’s environmental education, e.g., in middle school. 

## 6. Conclusions

The results of our study can help policymakers of Jinchuan mining area better understand the need for pollution information disclosure and education programs aimed at making local residents aware of air pollution and related health risks and enhancing their personal responses in reducing exposure to air pollutants. 

The present study is possibly the first on environmental knowledge and perception of air pollution in China, no prior information on measurement of these latent variables was available. Therefore, the indicators developed and tested here had not been tested before. Further development and testing of indicators of environmental knowledge and perception of air pollution is urgently needed, especially in the light of the alarming state of the environment in many Chinese cities. However, this kind of scientific research has a substantially wider application than just China because most cities in most developing countries are suffering from similar problems. The present paper could be a starting point for further theoretical and empirical research on the interaction between environmental knowledge and the perception of air pollution but also of other kinds of pollution in China. It could also stimulate similar research in other developing countries, in Western countries and be used for cross-national or cross-cultural comparisons. This study has also introduced notions that could be applied in studies on the perception of the probability of suffering from an illness and in averting behavior studies.

## Figures and Tables

**Table 1:** Descriptive statistics for the observed exogenous variables.

**Variables**			**Min**	**Max**		**Mean**	**S.D**	
**Age (AGE)**			21	78		44.11	11.4	
**Family Size (FS)**			1	6		2.95	0.78	
**Family health experience (FHE)**			0	1		0.33	0.48	
**Highest Level of Education (EDU)**		**Absolute**	**Percentage**	**Household Net Income (CNY per Month) (IN)**		**Absolute**	**Percentage**	
Primary school		48	(6.00%)	1000–2000		36	(4.70%)	
Middle school		179	(23.60%)	2000–3000		116	(15.30%)	
High school		192	(25.30%)	3000–4000		139	(18.30%)	
Vocational school		192	(25.30%)	4000–5000		145	(19.10%)	
Bachelor’s degree		145	(19.10%)	5000–6000		159	(20.90%)	
Master’s degree		3	(0.40%)	6000–7000		99	(13.00%)	
**Proximity to the Pollution Source (PPS)**				7000–8000		28	(3.70%)	
Nearby smelting plants, severe air pollution (SAP)	225		(29.60%)	8000–9000		14	(1.80%)	
				9000–10,000		8	(1.10%)	
Medium air pollution (MAP)	226		(29.80%)	More than 10,000		15	(2.00%)	
Far away from smelting plants, light air pollution (LAP, reference case)	308		(40.60%)					
**Work Environment (WE)**								
Non-JMC individual (reference case)	452		(59.55%)					
Miners and smelters worker of JMC (MS)	138		(18.18%)					
JMC employee, but not miner or smelter worker (NMS)	169		(22.27%)					

Notes: **Family size (FS)**: number of family members living in the same house; **Family Health experience (FHE)**: 1 if the respondent or one or more of their family members has been hospitalized for cardiovascular (e.g., hypertension, heart attack, chest pain, arrhythmia and myocardial infraction) or respiratory diseases (e.g., upper respiratory tract infection, bronchitis, pneumonia, asthma, and lung cancer), 0 otherwise. Source: Author’s survey.

**Table 2:** Overall goodness of fit indices.

Fit Index	Initial Model	Final Model	Cut off Value
Goodness-of-Fit Index (GFI)	0.98	0.98	>0.90
χ^2^/DF	2.53	2.47	<3.00
Comparative Fit Index (CFI)	0.91	0.93	>0.90
Incremental Fit Index (IFI)	0.92	0.93	>0.90
Adjusted Goodness-of-Fit Index (AGFI)	0.97	0.97	>0.80
Root Mean Square Error of Approximation (RMSEA)	0.045	0.041	<0.05

**Table 3:** The measurement models (standardized coefficients).

Latent Variables	Final Model				Latent Variables	Initial Model			
	Indicators	Coefficient	Standard Errors	*R*^2^		Indicators	Coefficient	Standard Errors	*R*^2^
**Perceived intensity of pollution (PAPL1)**	**PAP1**	1.00	-	1.00	**Perception of air pollution (PAP)**	**PAP1**	0.16	0.05	0.03
**Perceived hazardousness of the pollutants (PAPL2)**	**PAP2**	0.59	0.03	0.35		**PAP2**	0.60	0.04	0.35
	**PAP3**	0.53	0.03	0.28		**PAP3**	0.53	0.05	0.28
	**PAP4**	0.62	0.03	0.38		**PAP4**	0.62	0.04	0.38
	**PAP5**	0.55	0.03	0.30		**PAP5**	0.55	0.05	0.30
**Environmental knowledge (EK)**	**EK1**	0.49	0.04	0.24	**Environmental knowledge (EK)**	**EK1**	0.49	0.06	0.24
	**EK2**	0.43	0.04	0.19		**EK2**	0.43	0.06	0.19
	**EK3**	0.43	0.04	0.19		**EK3**	0.43	0.07	0.19
	**EK4**	0.51	0.03	0.25		**EK4**	0.50	0.07	0.25
	**EK5**	0.57	0.04	0.32		**EK5**	0.57	0.07	0.32
	**EK5**	0.51	0.03	0.26		**EK5**	0.51	0.07	0.26
	**EK7**	0.36	0.03	0.13		**EK7**	0.36	0.07	0.13
	**EK8**	0.44	0.03	0.19		**EK8**	0.44	0.07	0.19
**Socio-economic status (SES)**	Education	0.48	0.05	0.23	**Socio-economic status (SES)**	Education	0.45	0.05	0.21
	Income	0.46	0.04	0.21		Income	0.46	0.04	0.21

Note: All coefficients in the Final model and the Initial model are statistically significant at 1%.

**Table 4:** The structural model (standardized coefficients).

Variables	Final Model			Initial Model	
	EK	PAPL1	PAPL2	EK	PAP
**Perception of air pollution (PAP)**				0.22	
				(0.23)	
**Environmental knowledge(EK)**		0.13 ***	0.66 ***		0.50 ***
		(0.04)	(0.08)		(0.30)
**Socio-economic status (SES)**	0.33 ***	0.06	0.14 ***	0.22 ***	0.29 ***
	(0.07)	(0.05)	(0.08)	(0.11)	(0.10)
**Age(AGE)**	0.09 ***			0.08 **	
	0.03			(0.03)	
**Family size (FS)**			−0.08 ***		−0.09 ***
			(0.03)		(0.03)
**Family health experience (FHE)**			0.05 *		0.06 **
			(0.03)		(0.03)
Medium air pollution (MAP)		0.09 ***	0.06	−0.02	0.08 *
		(0.03)	(0.04)	(0.04)	(0.04)
Serious air pollution (SAP)		0.19 ***	0.06	0.05	0.09 **
		(0.03)	(0.04)	(0.04)	(0.04)
JMC employee, but not miner or smelter worker (NMS)	0.08			0.06	−0.03
	(0.05)			(0.05)	(0.05)
Miner or smelter worker of JMC (MS)	0.16 ***			0.12 **	0.02
	(0.04)			(0.05)	(0.06)
*R*^2^	0.13	0.05	0.51	0.19	0.37

Note: Standard errors in parenthesis. *, ** and ***: 10%, 5% and 1%, respectively.

**Table 5:** Standardized total and indirect effects (Final model).

Variables	Indirect Effect			Total Effect		
	EK	PAPL1	PAPL2	EK	PAPL1	PAPL2
**Environmental knowledge (EK)**					0.13 ***	0.66 ***
					(0.04)	(0.08)
**Family size (FS)**						−0.08 ***
						(0.03)
**Age (AGE)**		0.01 **	0.06 ***	0.09 ***	0.01 **	0.06 ***
		(0.01)	(0.02)	(0.01)	(0.02)	(0.03)
**Socio-economic status (SES)**		0.04 ***	0.02 ***	0.33 ***	0.10 ***	0.35 ***
		(0.02)	(0.06)	(0.07)	(0.05)	(0.09)
**Family health experience (FHE)**						0.05 *
						(0.03)
Medium air pollution (MAP)					0.09 ***	0.06
					(0.03)	(0.04)
Serious air pollution (SAP)					0.19 ***	0.06
					(0.03)	(0.04)
JMC employee, but not miner or smelter worker (NMS)		0.01	0.05	0.08	0.01	0.05
		(0.01)	(0.04)	(0.05)	(0.01)	(0.04)
Miner or smelter worker of JMC (MS)		0.02 **	0.10 ***	0.16 ***	0.02 **	0.10 ***
		(0.01)	(0.03)	(0.04)	(0.01)	(0.04)

Note: Standard errors in parenthesis. *, ** and ***: 10%, 5% and 1%, respectively.
